# Supplementary material for: Genome- and Transcriptome-Wide Association Studies Identify Susceptibility Genes and Pathways for Periodontitis
Source: Cells. 2022 Dec 23;12(1):70. doi: 10.3390/cells12010070 (PMC9818314; doi:10.3390/cells12010070)
Supplement: Supplementary file 1 [file cells-12-00070-s001.zip › Supplementary Material.pdf]

Table S1. Characteristics of periodontitis cases and controls

	Total		GLIDE		FinnGen	
	**Case**	**Control**	**Case**	**Control**	**Case**	**Control**
**N**	38,532	316,185	17,353	28,210	21,179	287,975
**Gender**						
**Male**	-		-		135,408	
**Female**	-		-		173,746	

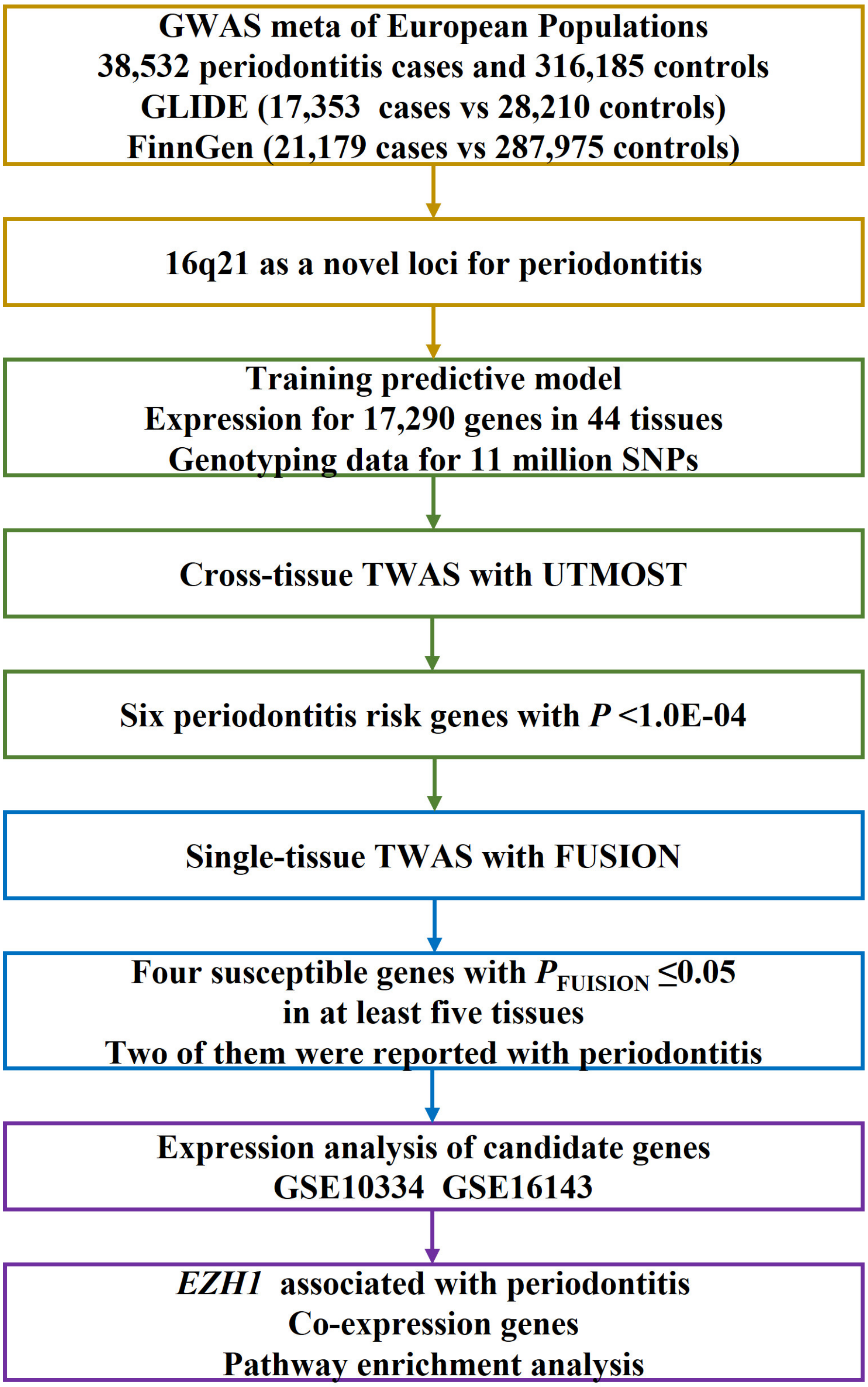

Figure S1. The flow chart of this study. GWAS, genome-wide association study; TWAS, transcriptome-wide association study; UTMOST, Unified Test for MOlecular SignaTures; eQTL, expression quantitative trait loci.

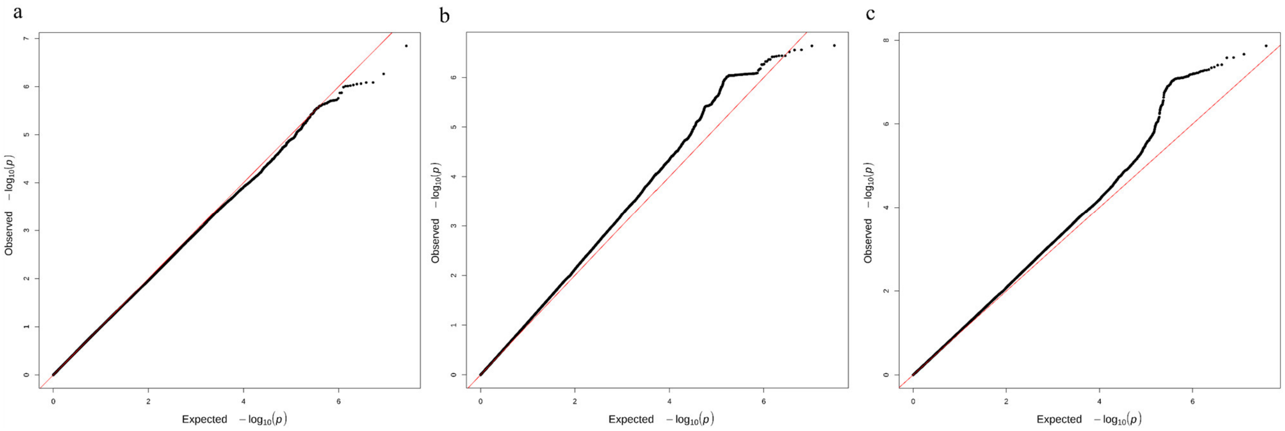

Figure S2. Quantile-quantile plot of -log10 (*P*-values) from all autosomal SNPs. (a) Summary data from GLIDE consortium (λ = 1.00). (b) Summary data from FinnGen (λ = 1.08). (c) Meta-analysis results (λ = 1.05).

Table S2. Significant SNPs of periodontitis in the GWAS

SNP	Chr	Position	Ref	Alt	Stage	OR (95% CI)	*P*
rs11076352	16	59988498	C	A	GLIDE	0.98 (0.97-0.99)	8.75E-04
					FinnGen	0.95 (0.93-0.97)	2.30E-07
					**Meta**	0.95 (0.93-0.97)	1.36E-08
rs28635813	16	60017097	T	C	GLIDE	0.98 (0.97-0.99)	1.01E-03
					FinnGen	0.95 (0.93-0.97)	5.47E-07
					**Meta**	0.95 (0.93-0.97)	2.15E-08
rs28394006	16	60017098	C	A	GLIDE	0.98 (0.97-0.99)	1.01E-03
					FinnGen	0.95 (0.93-0.97)	8.42E-07
					**Meta**	0.95 (0.94-0.97)	2.58E-08
rs11864657	16	60019808	G	A	GLIDE	0.98 (0.97-0.99)	1.59E-03
					FinnGen	0.95 (0.93-0.97)	8.81E-07
					**Meta**	0.95 (0.93-0.97)	2.62E-08
rs2406892	16	60026807	C	T	GLIDE	0.98 (0.97-0.99)	1.60E-03
					FinnGen	0.95 (0.93-0.97)	9.10E-07
					**Meta**	0.95 (0.94-0.97)	3.83E-08
rs12325580	16	59991400	C	T	GLIDE	0.98 (0.97-0.99)	9.05E-04
					FinnGen	0.95 (0.93-0.97)	3.83E-07
					**Meta**	0.95 (0.94-0.97)	3.92E-08
rs12922928	16	59984303	C	A	GLIDE	0.98 (0.97-0.99)	1.02E-03
					FinnGen	0.95 (0.93-0.97)	1.02E-06
					**Meta**	0.95 (0.94-0.97)	4.36E-08
rs2216750	16	60022166	G	A	GLIDE	0.98 (0.97-0.99)	9.98E-04
					FinnGen	0.95 (0.93-0.97)	8.29E-07
					**Meta**	0.95 (0.94-0.97)	4.54E-08

SNP, single nucleotide polymorphism; Chr, chromosome; Ref, reference allele; Alt, alternative allele; OR, odds ratio; 95% CI, 95% confidence interval.

Table S3. The significant results of FUSION for candidate susceptibility genes

Gene	Tissue	HSQ	BEST GWAS[a]	BEST eQTL[b]	MODEL[c]	SNP Number[d]	Z Score	*P*
SIGLEC14	Pancreas	0.26	rs16982907	rs16982907	top1	595	-4.91	9.34E-07
SIGLEC14	Prostate	0.15	rs16982907	rs16982907	top1	595	-4.91	9.34E-07
SIGLEC14	Small_Intestine_Terminal_Ileum	0.49	rs16982907	rs16982907	lasso	595	-4.74	2.13E-06
SIGLEC14	Skin_Not_Sun_Exposed_Suprapubic	0.45	rs16982907	rs7250849	lasso	595	-4.41	1.03E-05
SIGLEC14	Whole_Blood	0.36	rs16982907	rs7250849	enet	595	-4.39	1.15E-05
SIGLEC14	Artery_Aorta	0.37	rs16982907	rs16982907	lasso	595	-4.22	2.46E-05
SIGLEC14	Adipose_Visceral_Omentum	0.46	rs16982907	rs7250849	top1	595	-3.82	1.31E-04
SIGLEC14	Adrenal_Gland	0.53	rs16982907	rs7250849	top1	595	-3.82	1.31E-04
SIGLEC14	Artery_Coronary	0.20	rs16982907	rs7250849	top1	595	-3.82	1.31E-04
SIGLEC14	Artery_Tibial	0.41	rs16982907	rs7250849	top1	595	-3.82	1.31E-04
SIGLEC14	Brain_Amygdala	0.53	rs16982907	rs7250849	top1	594	-3.82	1.31E-04
SIGLEC14	Brain_Caudate_basal_ganglia	0.47	rs16982907	rs7250849	top1	595	-3.82	1.31E-04
SIGLEC14	Brain_Cortex	0.48	rs16982907	rs7250849	top1	595	-3.82	1.31E-04
SIGLEC14	Brain_Frontal_Cortex_BA9	0.45	rs16982907	rs7250849	top1	595	-3.82	1.31E-04
SIGLEC14	Brain_Hippocampus	0.63	rs16982907	rs7250849	top1	595	-3.82	1.31E-04
SIGLEC14	Brain_Hypothalamus	0.37	rs16982907	rs7250849	top1	593	-3.82	1.31E-04
SIGLEC14	Brain_Nucleus_accumbens_basal_ganglia	0.36	rs16982907	rs7250849	top1	595	-3.82	1.31E-04
SIGLEC14	Brain_Putamen_basal_ganglia	0.22	rs16982907	rs7250849	top1	595	-3.82	1.31E-04
SIGLEC14	Brain_Substantia_nigra	0.58	rs16982907	rs7250849	top1	594	-3.82	1.31E-04
SIGLEC14	Breast_Mammary_Tissue	0.40	rs16982907	rs7250849	top1	595	-3.82	1.31E-04
SIGLEC14	Cells_EBV-transformed_lymphocytes	0.37	rs16982907	rs7250849	top1	594	-3.82	1.31E-04
SIGLEC14	Colon_Sigmoid	0.36	rs16982907	rs7250849	top1	595	-3.82	1.31E-04
SIGLEC14	Colon_Transverse	0.33	rs16982907	rs7250849	top1	595	-3.82	1.31E-04
SIGLEC14	Esophagus_Gastroesophageal_Junction	0.40	rs16982907	rs7250849	top1	595	-3.82	1.31E-04
SIGLEC14	Heart_Atrial_Appendage	0.37	rs16982907	rs7250849	top1	595	-3.82	1.31E-04
SIGLEC14	Heart_Left_Ventricle	0.47	rs16982907	rs7250849	top1	595	-3.82	1.31E-04
SIGLEC14	Liver	0.37	rs16982907	rs7250849	top1	594	-3.82	1.31E-04
SIGLEC14	Muscle_Skeletal	0.21	rs16982907	rs7250849	top1	595	-3.82	1.31E-04
SIGLEC14	Stomach	0.31	rs16982907	rs7250849	top1	595	-3.82	1.31E-04
SIGLEC14	Thyroid	0.39	rs16982907	rs7250849	top1	595	-3.82	1.31E-04
SIGLEC14	Pituitary	0.33	rs16982907	rs16982912	lasso	595	-3.66	2.55E-04
SIGLEC14	Skin_Sun_Exposed_Lower_leg	0.27	rs16982907	rs7250849	lasso	595	-3.61	3.12E-04
SIGLEC14	Testis	0.41	rs16982907	rs7250849	lasso	595	-3.55	3.79E-04
SIGLEC14	Esophagus_Muscularis	0.50	rs16982907	rs16982912	lasso	595	-2.92	3.53E-03
SIGLEC14	Nerve_Tibial	0.57	rs16982907	rs7250849	enet	595	-2.92	3.55E-03
SIGLEC14	Brain_Spinal_cord_cervical_c-1	0.62	rs16982907	rs7250849	lasso	593	-2.84	4.52E-03
SIGLEC14	Lung	0.42	rs16982907	rs7250849	lasso	595	-2.52	1.19E-02
SIGLEC14	Esophagus_Mucosa	0.30	rs16982907	rs16982907	enet	595	-2.50	1.26E-02
SIGLEC14	Ovary	0.43	rs16982907	rs3786625	blup	595	-2.47	1.33E-02
SIGLEC14	Adipose_Subcutaneous	0.53	rs16982907	rs7250849	lasso	595	-2.35	1.89E-02
EZH1	Artery_Tibial	0.00	rs12943352	rs7208107	blup	268	-3.60	3.23E-04
EZH1	Breast_Mammary_Tissue	0.04	rs12943352	rs7217821	lasso	268	3.31	9.30E-04
EZH1	Brain_Putamen_basal_ganglia	0.10	rs12943352	rs8073311	blup	267	3.12	1.78E-03
EZH1	Heart_Atrial_Appendage	0.10	rs12943352	rs9766	top1	268	3.01	2.66E-03
EZH1	Pituitary	0.05	rs12943352	rs9766	top1	268	3.01	2.66E-03
EZH1	Esophagus_Mucosa	0.12	rs12943352	rs9766	top1	268	3.01	2.66E-03
EZH1	Brain_Cerebellar_Hemisphere	0.07	rs12943352	rs7209612	top1	267	2.93	3.37E-03

EZH1	Artery_Coronary	0.06	rs12943352	rs4792953	enet	268	2.86	4.18E-03
EZH1	Skin_Sun_Exposed_Lower_leg	0.09	rs12943352	rs9897724	top1	268	2.80	5.13E-03
EZH1	Adipose_Visceral_Omentum	0.03	rs12943352	rs2089115	top1	268	2.80	5.17E-03
EZH1	Skin_Not_Sun_Exposed_Suprapubic	0.20	rs12943352	rs2089115	top1	268	2.80	5.17E-03
EZH1	Esophagus_Muscularis	0.01	rs12943352	rs2089115	top1	268	2.80	5.17E-03
EZH1	Nerve_Tibial	0.09	rs12943352	rs2089115	top1	268	2.80	5.17E-03
EZH1	Thyroid	0.09	rs12943352	rs2089115	top1	268	2.80	5.17E-03
EZH1	Lung	0.04	rs12943352	rs7359598	top1	268	2.67	7.59E-03
EZH1	Brain_Cerebellum	0.22	rs12943352	rs2089116	top1	268	2.61	9.00E-03
EZH1	Brain_Spinal_cord_cervical_c-1	0.16	rs12943352	rs2089116	top1	268	2.61	9.00E-03
EZH1	Brain_Frontal_Cortex_BA9	0.07	rs12943352	rs1799967	blup	266	2.59	9.56E-03
EZH1	Cells_Transformed_fibroblasts	0.03	rs12943352	rs4796664	enet	267	2.58	9.75E-03
EZH1	Whole_Blood	0.08	rs12943352	rs9897724	enet	268	2.58	9.83E-03
EZH1	Testis	0.06	rs12943352	rs4792953	blup	268	2.37	1.79E-02
EZH1	Spleen	0.05	rs12943352	rs4792953	lasso	268	2.36	1.84E-02
EZH1	Adipose_Subcutaneous	0.12	rs12943352	rs9897724	lasso	268	1.97	4.85E-02
MRPS23	Brain_Hypothalamus	0.06	rs868194	rs17835083	lasso	544	-3.07	2.14E-03
MRPS23	Brain_Hippocampus	0.40	rs868194	rs4793891	blup	545	-2.86	4.22E-03
MRPS23	Pituitary	0.04	rs868194	rs11652711	top1	545	-2.39	1.70E-02
MRPS23	Heart_Left_Ventricle	0.06	rs868194	rs17835016	top1	545	2.37	1.77E-02
MRPS23	Testis	0.12	rs868194	rs17835016	lasso	545	2.09	3.70E-02
SIGLEC5	Cells_EBV-transformed_lymphocytes	0.27	rs16982907	rs16982907	top1	594	4.91	9.34E-07
SIGLEC5	Adrenal_Gland	0.25	rs16982907	rs11084094	enet	595	4.45	8.55E-06
SIGLEC5	Esophagus_Mucosa	0.09	rs16982907	rs875283	enet	595	4.28	1.85E-05
SIGLEC5	Artery_Aorta	0.09	rs16982907	rs4802828	enet	595	4.08	4.53E-05
SIGLEC5	Heart_Left_Ventricle	0.11	rs16982907	rs11667868	enet	595	3.99	6.68E-05
SIGLEC5	Brain_Cortex	0.01	rs16982907	rs11084094	top1	595	3.66	2.54E-04
SIGLEC5	Breast_Mammary_Tissue	0.09	rs16982907	rs11084094	top1	595	3.66	2.54E-04
SIGLEC5	Pituitary	0.03	rs16982907	rs11084094	top1	595	3.66	2.54E-04
SIGLEC5	Colon_Sigmoid	0.45	rs16982907	rs11084095	lasso	595	3.42	6.28E-04
SIGLEC5	Heart_Atrial_Appendage	0.36	rs16982907	rs11665668	enet	595	3.26	1.13E-03
SIGLEC5	Skin_Sun_Exposed_Lower_leg	0.15	rs16982907	rs11084095	blup	595	3.21	1.33E-03
SIGLEC5	Stomach	0.19	rs16982907	rs11084095	enet	595	3.18	1.49E-03
SIGLEC5	Lung	0.06	rs16982907	rs11084095	enet	595	3.13	1.76E-03
SIGLEC5	Nerve_Tibial	0.37	rs16982907	rs11084094	lasso	595	3.03	2.47E-03
SIGLEC5	Prostate	0.20	rs16982907	rs4802828	top1	595	2.45	1.45E-02
SIGLEC5	Esophagus_Gastroesophageal_Junction	0.11	rs16982907	rs8104955	enet	595	2.34	1.90E-02
SIGLEC5	Whole_Blood	0.37	rs16982907	rs7250849	lasso	595	-2.25	2.48E-02
SIGLEC5	Adipose_Subcutaneous	0.27	rs16982907	rs11084095	enet	595	2.17	2.99E-02
SIGLEC5	Testis	0.08	rs16982907	rs10853839	blup	595	2.15	3.19E-02
SIGLEC5	Adipose_Visceral_Omentum	0.24	rs16982907	rs11665668	lasso	595	1.97	4.91E-02
TMED7	Brain_Cerebellar_Hemisphere	0.06	rs173635	rs6887979	blup	522	-2.67	7.53E-03

HSQ, the heritability of the gene; [a] rsID of the most significant GWAS SNP in locus; [b] rsID of the best eQTL in the locus; [c] best performing model; [d] number of SNPs in the model.

Table S6. Top 10 diseases associated with the *EZH1* gene according to CTD

Disease Name	Inference Network	Inference Score	Reference Count
Inflammation	1,2-Dimethylhydrazine|4-(5-benzo(1,3)dioxol-5-yl-4-pyridin-2-yl-1H-imidazol-2-yl)benzamide|Acetaminophen|Air Pollutants|Amiodarone|Asbestos, Crocidolite|Ascorbic Acid|Benzo(a)pyrene|bisphenol A|Carbon Tetrachloride|Cisplatin|Cyclosporine|Dexamethasone|Doxorubicin|Estradiol|Isoproterenol|Ketamine|Mercury|Methapyrilene|Nanotubes, Carbon|Nickel|Nitric Oxide|Ozone|Paraquat|Particulate Matter|perfluorooctanoic acid|Progesterone|Resveratrol|Smoke|sodium arsenite|Sodium Selenite|Soot|Tetrachlorodibenzodioxin|Tetracycline|Thioacetamide|titanium dioxide|Trichloroethylene|Valproic Acid|Vehicle Emissions|Vitamin E|Water Pollutants, Chemical	140.01	214
Necrosis	1,2-Dimethylhydrazine|2,4-dinitrotoluene|2,6-dinitrotoluene|Acetaminophen|Amiodarone|Asbestos, Crocidolite|Ascorbic Acid|Benzo(a)pyrene|bisphenol A|Buthionine Sulfoximine|Carbon Tetrachloride|Chlorpromazine|Cisplatin|Cyclosporine|Dexamethasone|Diuron|dorsomorphin|Doxorubicin|Estradiol|Isoproterenol|Ketamine|Methapyrilene|Nanotubes, Carbon|Nickel|Nitric Oxide|Paraquat|Particulate Matter|perfluorooctane sulfonic acid|perfluorooctanoic acid|Resveratrol|sodium arsenite|Sodium Selenite|Tetrachlorodibenzodioxin|Tetracycline|Thioacetamide|titanium dioxide|Trichloroethylene|Valproic Acid|Vehicle Emissions|Vitamin E|Warfarin	136.05	343
Prenatal Exposure Delayed Effects	Air Pollutants|Amiodarone|Ascorbic Acid|Benzo(a)pyrene|bisphenol A|Chlorpromazine|Cisplatin|Cyclosporine|Dexamethasone|Dibutyl Phthalate|Endocrine Disruptors|Estradiol|Ethinyl Estradiol|Folic Acid|Mercury|Ozone|Paraquat|Particulate Matter|perfluoro-n-nonanoic acid|perfluorooctane sulfonic acid|perfluorooctanoic acid|Resveratrol|Smoke|sodium arsenite|Sodium Glutamate|Soot|Tetrachlorodibenzodioxin|titanium dioxide|Trichloroethylene|tris(1,3-dichloro-2-propyl)phosphate|Urethane|Valproic Acid|Vehicle Emissions|Warfarin	133	326
Chemical and Drug Induced Liver Injury	1,2-Dimethylhydrazine|2,4-dinitrotoluene|2,6-dinitrotoluene|Acetaminophen|Amiodarone|Ascorbic Acid|Benzo(a)pyrene|bisphenol A|Buthionine Sulfoximine|Carbon Tetrachloride|Chlorpromazine|Cisplatin|Cyclosporine|Dexamethasone|Dibutyl Phthalate|Doxorubicin|Ellagic Acid|Estradiol|Ethinyl Estradiol|Folic Acid|Mercury|Methapyrilene|Nanotubes, Carbon|Nickel|Nitric Oxide|Ozone|Paraquat|Particulate Matter|perfluoro-n-nonanoic acid|perfluorooctane sulfonic acid|perfluorooctanoic acid|Progesterone|Resveratrol|Smoke|sodium arsenite|Sodium Glutamate|Sodium Selenite|Tetrachlorodibenzodioxin|Tetracycline|Thioacetamide|titanium dioxide|Trichloroethylene|tris(1,3-dichloro-2-propyl)phosphate|Valproic Acid|Vehicle Emissions|Vitamin E|Warfarin	131.8	908

Weight Loss	1,2-Dimethylhydrazine|Acetaminophen|Air Pollutants|Amiodarone|Ascorbic Acid|Benzo(a)pyrene|bisphenol A|Carbon Tetrachloride|Cisplatin|Cyclosporine|Dexamethasone|Dibutyl Phthalate|Doxorubicin|Ellagic Acid|Estradiol|Folic Acid|Isoproterenol|Ketamine|Mercury|Methapyrilene|Paraquat|Particulate Matter|perfluoro-n-nonanoic acid|perfluorooctane sulfonic acid|perfluorooctanoic acid|Resveratrol|Smoke|sodium arsenite|Sodium Glutamate|Sodium Selenite|Tetrachlorodibenzodioxin|Thioacetamide|titanium dioxide|tris(1,3-dichloro-2-propyl)phosphate|Valproic Acid|Vehicle Emissions|Vitamin E|Water Pollutants, Chemical	131.71	192
Kidney Diseases	Acetaminophen|Ascorbic Acid|Benzo(a)pyrene|bisphenol A|Buthionine Sulfoximine|Carbon Tetrachloride|Cisplatin|Cyclosporine|Dexamethasone|Dibutyl Phthalate|Doxorubicin|Ellagic Acid|Estradiol|Ethinyl Estradiol|Folic Acid|Ketamine|Mercury|Nanotubes, Carbon|Nickel|Nitric Oxide|Ozone|Paraquat|Particulate Matter|Progesterone|Resveratrol|sodium arsenite|Sodium Selenite|Tetracycline|Thioacetamide|titanium dioxide|Trichloroethylene|Valproic Acid|Vehicle Emissions|Vitamin E|Warfarin|Water Pollutants, Chemical	114.89	365
Fibrosis	Acetaminophen|Air Pollutants|Amiodarone|AZM551248|bisphenol A|Carbon Tetrachloride|Cisplatin|Cyclosporine|Dexamethasone|Dibutyl Phthalate|Doxorubicin|Folic Acid|Isoproterenol|Ketamine|Methapyrilene|Nanotubes, Carbon|Paraquat|Particulate Matter|perfluorooctane sulfonic acid|Resveratrol|sodium arsenite|Tetrachlorodibenzodioxin|Thioacetamide|titanium dioxide|Trichloroethylene|trichostatin A|Valproic Acid|Vehicle Emissions	109.48	93
Hyperplasia	1,2-Dimethylhydrazine|4-(5-benzo(1,3)dioxol-5-yl-4-pyridin-2-yl-1H-imidazol-2-yl)benzamide|Acetaminophen|Asbestos, Crocidolite|Ascorbic Acid|Benzo(a)pyrene|bisphenol A|Carbon Tetrachloride|Cyclosporine|Dexamethasone|Dibutyl Phthalate|Diuron|Ellagic Acid|Estradiol|Ethinyl Estradiol|Isoproterenol|Methapyrilene|Nanotubes, Carbon|Nitric Oxide|Particulate Matter|perfluorooctanoic acid|Resveratrol|sodium arsenite|Tetrachlorodibenzodioxin|Tetracycline|Thioacetamide	99.26	59
Nerve Degeneration	Air Pollutants|Ascorbic Acid|Benzo(a)pyrene|bisphenol A|Cisplatin|Cyclosporine|Dexamethasone|Doxorubicin|Estradiol|Isoproterenol|Ketamine|Mercury|Nickel|Nitric Oxide|Paraquat|Particulate Matter|perfluorooctane sulfonic acid|perfluorooctanoic acid|Progesterone|Resveratrol|sodium arsenite|Sodium Glutamate|Trichloroethylene|trichostatin A|Urethane|Valproic Acid|Vehicle Emissions|Vitamin E	96.41	83
Cell Transformation, Neoplastic	2,6-dinitrotoluene|Asbestos, Crocidolite|Ascorbic Acid|Benzo(a)pyrene|bisphenol A|Buthionine Sulfoximine|Cisplatin|Cyclosporine|Doxorubicin|Ellagic Acid|Estradiol|Methapyrilene|Nanotubes, Carbon|Nickel|Particulate Matter|perfluorooctane sulfonic acid|perfluorooctanoic acid|Resveratrol|Smoke|sodium arsenite|Thioacetamide|titanium dioxide|Vanadates|Vehicle Emissions|Vitamin E|Water Pollutants, Chemical	96.28	97

CTD, the Comparative Toxicogenomics Database. References can be accessed via hyperlinks.

Table S7. Top 10 GO and KEGG terms identified by enrichment analysis for genes co-expressed with *EZH1*

ID	Term	GeneRatio	P_{value}	P_{adjust}
GO:0030055	cell-substrate junction	181/4991	1.35E-14	1.04E-11
GO:0005925	focal adhesion	176/4991	6.56E-14	2.53E-11
GO:0005774	vacuolar membrane	184/4991	2.71E-13	6.97E-11
GO:0005765	lysosomal membrane	168/4991	6.83E-13	1.05E-10
GO:0098852	lytic vacuole membrane	168/4991	6.83E-13	1.05E-10
GO:0016607	nuclear speck	170/4991	8.59E-13	1.11E-10
GO:0031252	cell leading edge	167/4991	2.49E-11	2.74E-09
GO:0031983	vesicle lumen	132/4991	1.97E-09	1.90E-07
GO:0060205	cytoplasmic vesicle lumen	131/4991	2.53E-09	2.17E-07
GO:0034774	secretory granule lumen	129/4991	5.22E-09	4.03E-07
KEGG: hsa05169	Epstein-Barr virus infection	96/2339	6.91E-09	9.93E-07
KEGG: hsa04142	Lysosome	69/2339	7.61E-09	9.93E-07
KEGG: hsa04141	Protein processing in endoplasmic reticulum	84/2339	8.92E-09	9.93E-07
KEGG: hsa04910	Insulin signaling pathway	70/2339	2.04E-08	1.70E-06
KEGG: hsa04662	B cell receptor signaling pathway	47/2339	4.74E-08	3.17E-06
KEGG: hsa04722	Neurotrophin signaling pathway	58/2339	2.44E-06	1.33E-04
KEGG: hsa05165	Human papillomavirus infection	133/2339	2.78E-06	1.33E-04
KEGG: hsa05166	Human T-cell leukemia virus 1 infection	95/2339	3.51E-06	1.47E-04
KEGG: hsa04151	PI3K-Akt signaling pathway	140/2339	4.23E-06	1.57E-04
KEGG: hsa04931	Insulin resistance	53/2339	5.08E-06	1.58E-04

GO, Gene Ontology; KEGG, Kyoto Encyclopedia of Genes and Genomes; GeneRatio, genes of interest in the gene set / total genes of interest.
